# A Self-Healing and Electrical-Tree-Inhibiting Epoxy Composite with Hydrogen-Bonds and SiO_2_ Particles

**DOI:** 10.3390/polym9090431

**Published:** 2017-09-08

**Authors:** Wancong Bian, Wenxuan Wang, Ying Yang

**Affiliations:** Department of Electrical Engineering, Tsinghua University, Beijing 100084, China; bwc16@mails.tsinghua.edu.cn (W.B.); KillerHusky@126.com (W.W.)

**Keywords:** electrical self-healing, electrical tree, epoxy composite, insulation

## Abstract

Electrical tree growth in the insulation material is a main factor limiting the lifespan of insulation. A new method of increasing the durability and reliability of polymer dielectrics has been proposed by designing a three-phase electrical self-healing composite. SiO_2_ micro and nano particles were loaded in the sample which can improve the resistance to electrical tree breakdown. Materials with hydrogen bonds were synthesized and added into epoxy matrix to make the composite self-healable. It is found that both SiO_2_ and hydrogen-bonding self-healing material (HSM) can inhibit the electrical trees. Besides the self-healing behavior at the macro level, the incorporation of HSM can also make the micro defects such as electrical tree channel self-healable. The electrical self-healing composite will find a wide application in the field of electronic and electrical engineering.

## 1. Introduction

Dielectric materials are the indispensable elements in microelectronics and high voltage electrical devices in insulation and packaging. Polymers are preferred dielectric materials owing to their ease of processing, tunable dielectric constants, and low cost. Polymer degradation and electrical tree growth arise from the creation of intense electric fields at the tips of conducting inhomogeneity, discharge channels and sites of stress concentration [1]. The intense electric field leads to localized breakdown, creating cavities and tree channels [2] which influence the long-term degradation of the polymer dielectrics. If the growth of these electrical trees could be avoided, the lifetime and reliability of the insulation could be greatly increased. The evolution toward electronics and electrical devices with high reliability of power densities with smaller feature sizes and long lifetime places high demands on a set of material properties, especially the electrical, and self-healing properties, of polymer dielectrics.

The ideal insulting polymer dielectrics should be a homogenous one with no defects in the bulk. However, the homogenous bulk shows limitations in insulating materials properties. Loading additives to enhance the performance of polymer dielectrics has attracted widespread attention [3,4]. The big volume of the micro particles and the large surface area of the nano particles are both found to contribute to enhancing the resistance to an electrical tree. Nanoparticles such as SiO_2_ and Al_2_O_3_ with different shapes were applied to form heterogeneous materials to increase the phase interface regions then enhance the electrical properties. Zhou et al. [5] reported that the mechanical properties of the composites may restrain the electrical aging process, especially limiting the development of partial discharges to form micro-size electrical trees in the bulk. Although there are many researches on the electrical tree behaviors of the micro composites, nano composites and the comparison of two, few has investigated into the resistance to electrical tree of the micro-nano composites containing both micro and nano particles.

Self-healing materials are currently of great interest because of the possibility of improving the reliability, durability, and lifetime of materials. Epoxy resins have a wide range of applications in electrical and electronics devices for insulating. They are more brittle than thermoplastics and are prone to crack. To overcome this obstacle, the concept of self-healing epoxy resins was proposed. There are three major categories of self-healing epoxy-based systems [6]: (i) capsule based [7,8,9,10]; (ii) vascular based [11,12,13,14,15]; (iii) intrinsic [16,17,18,19,20]. The third system can be achieved via Diels-Alder reaction [17], Hydrogen bonding [18] and ionic bonding [19], and thermoplastic phase [20]. An ideal self-healing material is capable of continuously sensing and responding to damage over the lifetime of the polymeric components and restoring the material’s performance without negatively affecting the initial material properties. The use of self-healing agents in insulation materials must consider the self-healing property of the material and the effects of the surface coating of the self-healing agents used to improve the compatibility of the agents with the intrinsic material since surfactants always introduce more interfaces which may negatively affect the insulation. Hollow fibers or hollow capsules appear in the material after the self-healing process, which introduce new defects which increase the electrical conductivity. In addition, the exhaustion of the healing agent in these self-healing materials means the loss of the self-healing ability. Therefore, the improvement of self-healing durability of the thermoset epoxy is the most challenging work in this field.

In this paper, three-phase electrical self-healing epoxies were prepared. SiO_2_ micro and nano particles were loaded in the sample which can improve the resistance to electrical tree breakdown. Hydrogen-bonding self-healing material (HSM) with different chain length were synthesized and added into epoxy matrix. The resistance to electrical tree were evaluated with different micro/nano SiO_2_ particles and HSM loading mass ratios. The dielectric parameters and volume resistivity are measured to study the interfacial self-healing of the composites. The electrical tree growth in the composites with low particles concentration (0.1 wt %) are observed under a stereomicroscope to study the effects of the SiO_2_ particles on enhancing resistance to electrical tree. The self-healing of the structural and electrical properties of the polymer nanocomposites after multiple fractures has been examined.

## 2. Materials and Methods

### 2.1. Materials

**Figure 1:**
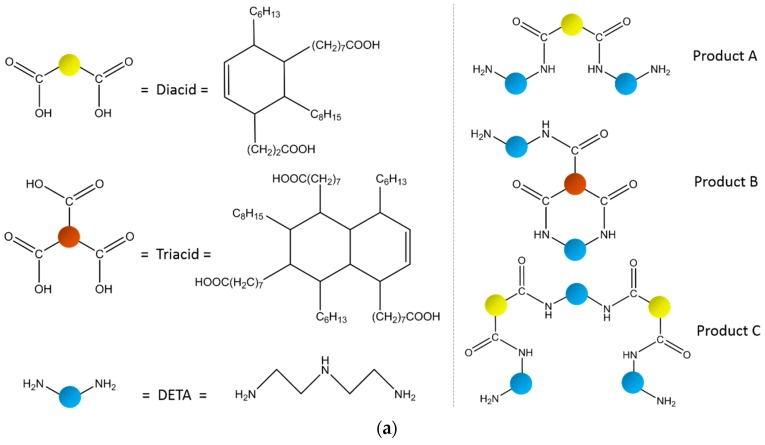

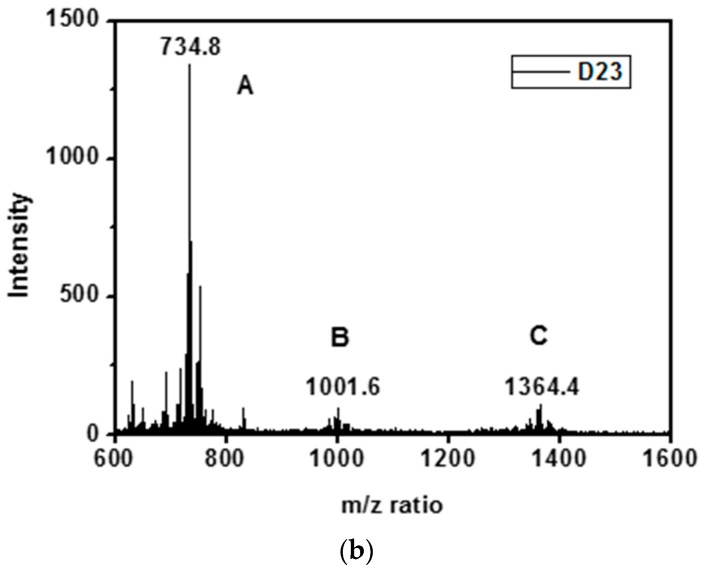
(**a**) The chemical structures of the three main products A, B and C in the HSM D23; (**b**) The MS of the products in the HSM D23. D34 and D45 were shown in Supplementary Figure S2.

The bicomponent liquid epoxy (component A, Bisphenol-A epoxy resin with an epoxy equivalent weight of 196 g/eq; component B, modified alicyclic amine curing agent. The stoichiometric ratio of the component A and component B was 2:1) used in this paper was a commercial product HT-6308 supplied from Shanghai Huitian new material Co., Ltd. (Shanghai, China). Diethylenetriamine (DETA), triethylenetetramine (TETA) and tetraethylenepentamine (TEPA) were purchased from Xiya Reagent Co., Ltd. (Chengdu, China) and ATUREX-1017 (80 wt % diacids, 17 wt % triacids, Figure 1) was supplied by Jiangxi Aturex Industrial Co., Ltd. (Yichun, China). Micro SiO_2_ with an average diameter of 20 μm and nano SiO_2_ with an average diameter of 30 nm were obtained from Beijing DK Nano Technology Co., Ltd. (Beijing, China) and silane coupling agent KH-550 was purchased from Kunshan Lvxun Electronic Material Co., Ltd. (Kunshan, China).

### 2.2. Preparation of HSM

The HSM was obtained through the amidation reaction of ATUREX-1017 and amines. In brief, 87.5 g of ATUREX-1017 was mixed with 35.2/49.9/64.6 g of DETA/TETA/TEPA and the mixtures were mechanically stirred at 160 °C under nitrogen for 24 h.

### 2.3. Surface Modification of SiO_2_ Particles

Micro SiO_2_ and nano SiO_2_ particles were modified with silane coupling agent KH-550. Briefly, 0.6 g of KH-550 was dissolved in 200 mL 95% alcohol under stirring for 5 min, then 6 g of micro SiO_2_ or nano SiO_2_ particles were added into the solution and the mixture was stirred under room temperature for 2 h. Finally, the modified SiO_2_ particles were collected by vacuum filtration and dried at 70 °C for 24 h and 130 °C for 12 h.

### 2.4. Preparation of Epoxy/HSM/SiO_2_ Composites

The epoxy/HSM/SiO_2_ composites were prepared by solution blending method. The HSM was first dissolved in chloroform at 80 °C for 20 min and the SiO_2_ particles were dispersed in chloroform and treated with ultrasound for 3 min at room temperature. A predetermined amount of epoxy, HSM and SiO_2_ were blended in chloroform and stirred for 5 min, followed by degassing for 30 min at 40 °C. Then, the mixture was precured for 2 h at 80 °C and postcured for 12 h at 100 °C according to a standard curing procedure for this epoxy system.

### 2.5. Characterization

The mass spectrum (MS) experiments of three kinds of HSM were carried out on a AXIMA Performance MALDI-TOF Mass Spectrometer (Shimadzu, Kyoto, Japan). Standard solutions were prepared at a concentration of 1 mg/L in ethanol. About 5 μL of these mixtures were applied in the sample tube, ionizated, and the spectra were recorded in positive reflectron ion mode.

The scanning electron microscope (SEM) unit (Hitachi SU8000, Tokyo, Japan) was used to analyze the fracture surface characteristics of the epoxy/HSM/SiO_2_ composite. The samples were coated with platinum before observation. The energy dispersive spectrometer (EDS) was used to analyze the element distribution on the fracture surface.

Thermogravimetric analysis (TGA) were performed on a Q500 (TA Instruments, New Castle, DE, USA) apparatus at a heating rate of 10 °C/min under nitrogen from 30 to 600 °C. 

For the thermally stimulated current (TSC) experiments, the samples were first polarized under 300 V/mm at 25 °C for 30 min and cooled down to −20 °C at a rate of −10 °C/min. Then the samples were depolarized for 10 min to release the polarization charges. Finally, the samples were linearly heated at 3 °C/min and the depolarization current was recorded. The samples were 2 mm thick and 20 mm in diameter.

The dielectric constants and the dielectric losses of the composites were measured by a broadband dielectric spectrometer Concept 40 (Novocontrol Technologies, Montabaur, Germany) with 1 V from 1 Hz to 1 MHz at 25 °C. The samples were 2 mm thick and 20 mm in diameter. To investigate the healing ability of dielectric parameters, two sheets of composite samples were stacked together at 80 °C for 10 min followed by cooling down with 2 kPa pressure for 5 min. The dielectric parameters of the sheets separately and together were measured.

The volume resistivity was measured using the three electrodes method, where the auxiliary electrode was used to shield the surface current. The resistivity was calculated according to Ohm’s law from the current after applying 1 kV direct voltage for 10 min. The samples were 1.5 mm thick and 70 mm in diameter. To investigate the healing ability of volume resistivity, two sheets of composite samples were stacked together at 80 °C for 10 min followed by cooling down with 2 kPa pressure for 5 min. The volume resistivity of the sheets separately and together were measured.

Mechanical tensile testing was performed using a vertical tensile tester (the original space between the clamps was 20 mm and the rate was 50 mm/min). 30 × 10 × 1.5 mm^3^ samples were exposed to a vertical force at room temperature. The self-healing experiments were performed by bringing severed samples together at room temperature. The samples were then heated in a vacuum drying oven at 80 °C for 2 h as a self-healing treatment.

### 2.6. Electrical Treeing Experiments

The cubic samples with pre-embedded steel needle electrode were prepared by curing and sized by a tetrafluoroethylene mold (Supplementary Figure S1). A piece of black conductive rubber was penetrated by a 0.16 mm diameter needle electrode and embedded at one side of the sample to provide a conductive contact with the external electrode. The tip of the needle electrode was located near the other side of the 30 × 28 × 3 mm^3^ cubic sample. The distance between the tip and the grounding surface is 2 mm (1 mm for electrical tree breakdown testing).

The electrical treeing experiments were performed with alternating voltage, the samples were clamped between a high voltage plate electrode and a ground plate electrode which were in good contact with the needle electrode and grounding surface of the samples, respectively. The AC high voltage of 15 kV (rms value) was applied to the samples. The development of the electrical tree was observed under a stereomicroscope at 100× magnification.

## 3. Results and Discussion

### 3.1. Material Characterization

The HSM labelled as D23, D34 and D45 were obtained through chemical reactions of a mixture of diacid and triacid with DETA/TETA/TEPA, respectively. The acids and amines are connected by amide bonds which provide N–H and C=O bonds to form hydrogen bonds between molecules. Figure 1a shows the chemical structures of the three kinds of products A, B and C in the HSM D23. The MS was used to identify the probable structures of the compound [21]. Figure 1b gives the MS of the HSM D23. The A peak (734.8 amu) in the first group was due to the chemical reactions between the diacid (564.9 amu) and DETA (103.2 amu) (1:2) after losing two H_2_O molecules (18.0 amu). The B peak (1001.6 amu) in the second group was obtained through reactions of the triacid (847.4 amu) with DETA (1:2) after losing three H_2_O molecules. The C peak (1364.4 amu) in the third group was obtained through reactions of the diacid with DETA (2:3) after losing four H_2_O molecules. Supplementary Figure S2 showed the MS and the chemical structures of the HSM D34 and D45. The MS results confirmed the chemical structures of the HSM products obtained through the amidation reaction of acids with amines.

**Figure 2:**
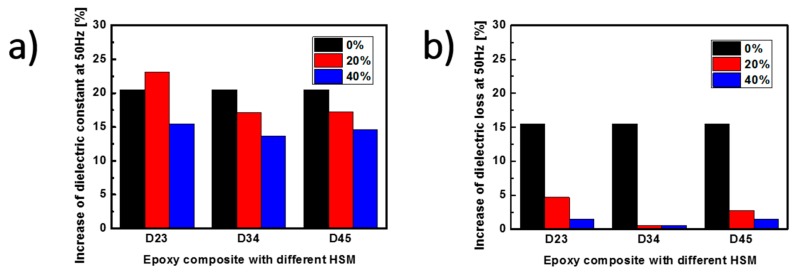
The increment of the (**a**) dielectric constants and (**b**) dielectric losses at 50 Hz of the three kinds of hydrogen-bonding self-healing material (HSM) D23, D34 and D45 after self-healing.

The epoxy/HSM composites with 20 and 40 wt % of three kinds of HSM were prepared and tested to select out the HSM with the best self-healing ability. Dielectric parameters are sensitive to the defects in the tested material. The heterogeneous interface between the two sheets of composite material samples will markedly increase the values of the dielectric parameters. A self-healable material should be able to lower this kind of dielectric parameters increase by healing the surface structures of the material at the interface. The dielectric constants and the dielectric losses of the composites are measured to distinguish the difference between the original separated samples before self-healing and the stacked samples after self-healing. Both the dielectric constants and the dielectric losses decrease for all the samples with increasing frequency mainly due to the dielectric relaxation [22]. The dielectric parameters of the composites with the same HSM wt % are close but increase when the HSM wt % increase from 20 to 40 wt % (Supplementary Figure S3). For example, the values of the dielectric constants and the dielectric losses at 1 kHz before self-healing are listed. The original samples with 20 wt % D23, D34, D45 have dielectric constants of 4.07, 4.46 and 4.45 with dielectric losses of 0.0298, 0.0355, and 0.0348. The original samples with 40 wt % D23, D34, D45 have dielectric constants of 5.95, 6.69 and 6.16 with dielectric losses of 0.141, 0.168 and 0.140. The self-healing ability is characterized by the increment of the values of the dielectric parameters at the power frequency of 50 Hz. As shown in Figure 2a,b, both the increment of the dielectric constant and the dielectric loss decrease with the higher wt % of HSM. The increment of the dielectric constant and the dielectric loss of the epoxy/D34 composite are the smallest. After self-healing, the dielectric constants of the epoxy/HSM composites with 0, 20, 40 wt % D34 increased 20.5%, 17.1%, 13.7% and the dielectric losses increased 15.5%, 0.5%, 0.5%. Thus, the HSM D34 was chosen as the self-healing additive in the epoxy/HSM/SiO_2_ composite system.

The glass transition temperature (*T*_g_) of the epoxy, HSM and epoxy/HSM composite with 40 wt % HSM were measured by Differential Scanning Calorimetry (DSC). The *T*_g_ of epoxy is 91.6 °C and that of the HSM D34 is −9.3 °C. Only one glass-transition point at 34.2 °C was observed in the DSC curve (Supplementary Figure S4), which indicated good compatibility between the HSM and the epoxy. Some of the amides in the HSM might react with the epoxy group, forming weak bonds between the HSM and the epoxy matrix.

**Figure 3:**
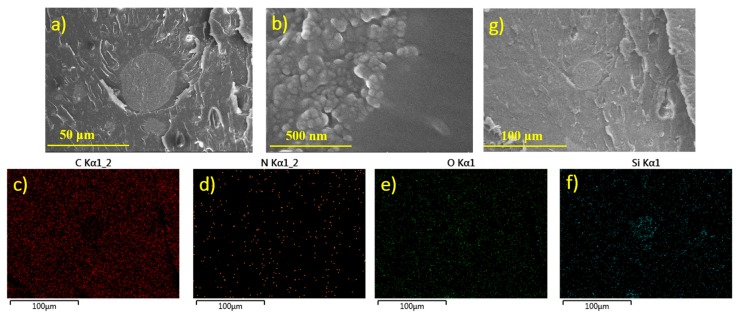
The SEM images of (**a**) the micro and (**b**) the nano SiO_2_ particles in the epoxy/HSM/SiO_2_ composites; The energy dispersive spectrometer (EDS) images of the elements (**c**) C, (**d**) N, (**e**) O and (**f**) Si, (**g**) The SEM image of the area being analyzed.

The SEM images of the micro and nano SiO_2_ particles in the epoxy/HSM/SiO_2_ composites are shown in Figure 3a,b. The size of the micro particles is 10~30 μm and the size of the nano particles is 20~50 nm, which almost match the given size of 20 μm and 30 nm. Figure 3b shows the edge of the micro SiO_2_ particle which is in the middle of Figure 3a. When the edge of the micro particle is zoomed in, the composite structure is found to be the micro particle covered by the nano particles as shown in Figure 3b. EDS was used to analyze the element distribution of the area shown in Figure 3g. The EDS images of the elements C, N, O and Si are shown in Figure 3c–f. Element C is much less in a middle circle area due to the existence of a micro SiO_2_ particle. The distribution of element N indicates that the HSM which contains amide bonds was uniformly dispersed in the composite system. The distribution of element O is less characteristic because all of epoxy, HSM and SiO_2_ contain element O. The distribution of element Si shown in Figure 3f shows an opposite pattern to element C. The density of Si is much higher in a middle circle area, which confirm that round particle to be micro SiO_2_ particle. Besides the micro SiO_2_ in the middle, nano SiO_2_ particles are also found uniformly dispersed in the composite system as shown in Figure 3f. From these images it can be inferred that the synthesized epoxy/HSM/SiO_2_ composites are uniform compounds.

### 3.2. Electrical Treeing Experiments

**Figure 4:**
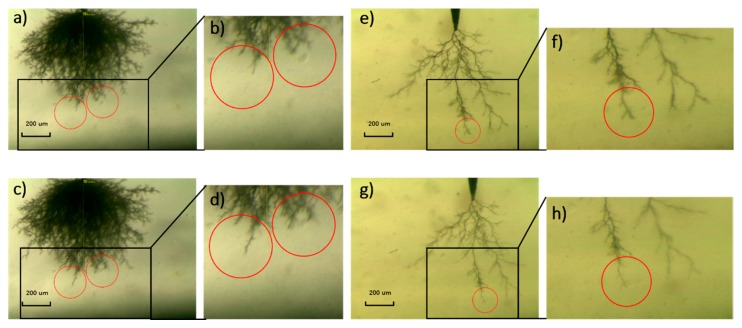
The electrical tree (**a**,**b**) reaches a micro SiO_2_ particle and then (**c**,**d**) bypass it; The small branches of the electrical tree (**e**,**f**) come into being and (**g**,**h**) decrease after self-healing.

Many researches have declared that the particles in the composites blocks the tip of the electrical tree so that electrical tree turns aside and the growth path of the electrical are hindered [23,24,25,26], but few researches gave a direct graphic evidence of the hindrance effect of the particles. As shown in Figure 4b,d, while the tree in the left circle can grow ahead more freely without any obstruction, the existence of a micro SiO_2_ particle hinders the electrical tree in the right circle so the tree path has to branch and bypass the particle. This phenomenon of electrical tree bypass induced by the particle hindrance consumes more energy provided from the electrical field, which means the electrical tree will grow with more difficultly when encountering the SiO_2_ particles. 

Reported researches on enhancing the resistance of the material to the electrical tree mostly focused on slowing down the speed of the tree growth. Here, a new method of self-healing is proposed to improve the resistance to the electrical treeing. As shown in Figure 4e–h, the trunk width became narrower and the number of small branches decreased. It indicates that the electrical tree channel can be healed after the self-healing process. Electrical treeing is caused by high pressure gas generated by the material decomposition [27] which expands the material to create the cavity and thin branches. The epoxy/SiO_2_/HSM composite was able to heal these micro defects caused by the mechanical stress through hydrogen bonds. The polymer degradation caused the accumulation of the conductive carbon in the electrical tree channel, which made the channel look black.

The electrical trees in several composites were analyzed, each test of one composite was repeated 10 times. The neat epoxy is labelled as EP. The epoxy/HSM/SiO_2_ composites are labelled as C0.1-D20 (0.1 wt % SiO_2_, micro SiO_2_:nano SiO_2_ = 1:3; 20 wt % HSM D34) and C0.1-D40 (0.1 wt % SiO_2_, micro SiO_2_:nano SiO_2_ = 1:3; 40 wt % HSM D34). As controls, the composites with only SiO_2_ or HSM are also prepared. The epoxy/SiO_2_ composites are labelled as C0.03-4/0 (0.03 wt % SiO_2_, micro SiO_2_:nano SiO_2_ = 4:0) and C0.1-0/4 (0.1 wt % SiO_2_, micro SiO_2_:nano SiO_2_ = 0:4). The epoxy/HSM composites are labelled as D34-20 (20 wt % HSM D34) and D34-40 (40 wt % HSM D34).

**Figure 5:**
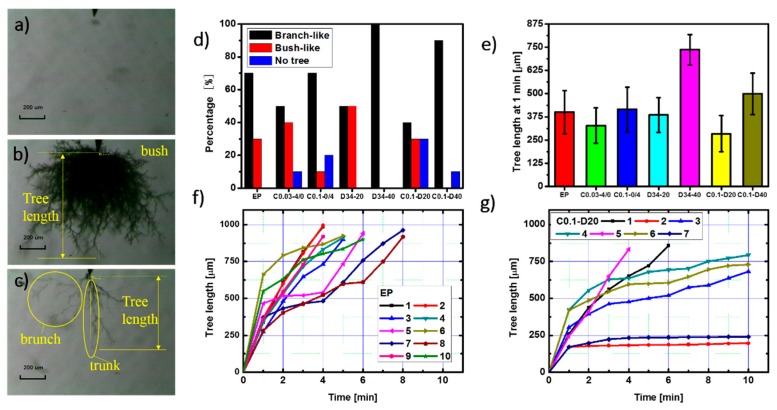
The images of (**a**) no tree; (**b**) bush-like tree and (**c**) branch-like tree; (**d**) The percentages of the three electrical tree shapes of the different composites; (**e**) The tree length at 1 min of the different composites; The tree lengths of (**f**) EP and (**g**) C0.1-D20 at different treeing time.

As shown in Figure 5a–c, the electrical tree shapes in the composites can be categorized as three kinds: no tree, bush-like tree and branch-like tree. Figure 5d shows the percentages of tree shape in the different composites. As for C0.1-D20 and C0.1-D40, the percentages of no tree are 30% and 10%, both are higher than 0% of EP. By comparing the percentages of no tree in epoxy/SiO_2_ composites and epoxy/HSM composites, it can be concluded that the incorporation of SiO_2_ will inhibit the tree inception, which lead to the no tree in the composites. Besides, the inhibiting effect of the nano SiO_2_ particles is stronger than micro SiO_2_ particles. Branch-like tree is more harmful than bush-like tree, because the electrical energy focuses on the tip of the branch-like tree, which usually makes the electrical trees grow more easily and faster. The percentages of branch-like tree are 50% and 100% in D34-20 and D34-40, which means that the incorporation amount of HSM should be moderate. The percentage of branch-like tree is 40% in C0.1-D20 which is the lowest among all the composites. It indicates that both SiO_2_ and HSM can inhibit the electrical trees. As shown in Figure 5e, after 1 min, the tree length of C01-D20 is 284 μm, 29% shorter than 401 μm of EP. Figure 5f,g shows how the electrical tree length grows with the increasing treeing time. If no tree grows in a sample, then the tree length of this sample will not be plotted. In general, the relation between tree length and time is nearly linear in EP and all the trees in EP reach 875 μm between 4 and 8 min. The epoxy/HSM/SiO_2_ composite C0.1-D20 shows a better resistance to electrical tree: 2 trees do not reach 250 μm, 3 trees do not reach 800 μm, and no tree is found in 3 samples after 10 min. The trees in the epoxy/HSM/SiO_2_ composite start to show a tendency to stabilize the tree length growth curves, which is due to the blocking effect of the micro particles, the inhibition effect of the nano particles and the moderate HSM.

After electrical treeing experiments, the samples of EP, C0.1-D20 and C0.1-D40 are heated at 80 °C for 2 h as self-healing treatment. Then, re-treeing experiments are conducted on the samples to test the treeing properties after self-healing.

**Figure 6:**
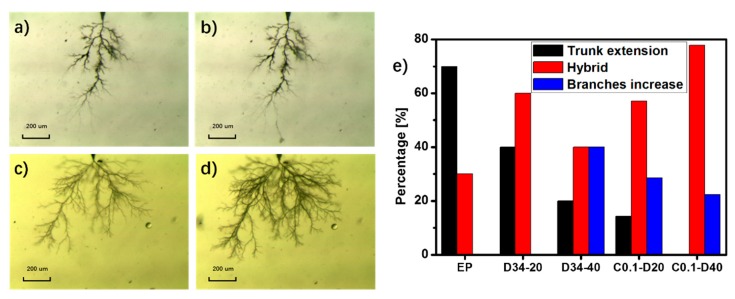
The electrical tree (**a**) before and (**b**) after the trunk extension; The electrical tree (**c**) before and (**d**) after the branches increase; (**e**) The percentages of the three re-treeing cases of the different composites.

There are three main cases of the re-treeing: trunk extension, branches increase and hybrid. As shown in Figure 6b, after re-treeing, the tree trunk extends forward compared the original tree in Figure 6a. As shown in Figure 6d, after re-treeing, the number of the branches increase compared the original tree in Figure 6c. Hybrid means both the trunk extension and the branches increase exist in the re-treeing sample. Trunk extension increase the tree length, which directly shortens the life of the insulation material. Electrical energy is shared by more treetops when branches increase, which lowers the probability of the further tree length extension. Figure 6e gives the percentages of the three re-treeing cases of the different composites. It can be seen that the percentage of trunk extension decreases with the increasing wt % HSM in the epoxy/HSM composites. As for D34-40 with 40 wt % HSM, the percentage of branches increase is 40% while that of EP is 0%. It indicates that besides the self-healing behavior at the macro level, the incorporation of HSM can also make the micro defects such as electrical tree channel self-healable. After self-healing, the electrical tree tends to find a new path rather than grow along the original way. As for the epoxy/HSM/SiO_2_ composites, the percentages of the trunk extension are 14% and 0%. It should be also noticed that the percentage of the branches increase of C0.1-D20 is 29% while that of D34-20 is 0%. These results indicate that the incorporation of SiO_2_ will help block and inhibit the tree trunk extension and induce the tree to grow sideways.

**Figure 7:**
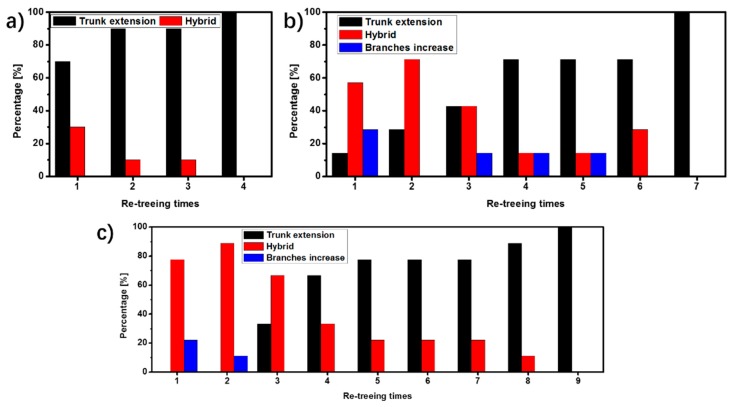
The percentages of the three re-treeing cases of (**a**) EP, (**b**) C0.1-D20 and (**c**) C0.1-D40 after different re-treeing times.

The percentages of the re-treeing cases in EP, C0.1-D20 and C0.1-D40 are shown in Figure 7. As for EP, the percentage of trunk extension exceeds 50% after the 1st re-treeing and reaches 100% after the 4th re-treeing. Both the percentages of trunk extension in C0.1-D20 and C0.1-D40 exceed 50% after the 4th re-treeing. The percentage of trunk extension in C0.1-D20 and C0.1-D40 reaches 100% after the 7th and the 9th re-treeing, respectively. This indicates that the self-healing cycles of the epoxy/HSM/SiO_2_ composites increase with the increasing amount of HSM.

### 3.3. Further Analysis of the Electrical Treeing

**Figure 8:**
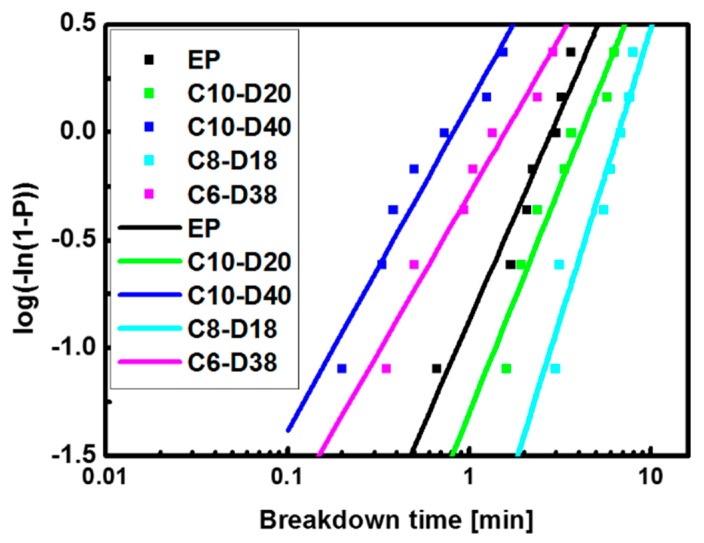
The Weibull distribution of the breakdown time of the epoxy/HSM/SiO_2_ composites.

The epoxy/HSM/SiO_2_ composites with higher wt % SiO_2_ were also prepared for electrical treeing experiments. The composites are labelled as CX-DY, which means there are X wt % SiO_2_ particles (micro SiO_2_:nano SiO_2_ = 1:3) and Y wt % HSM D34. Because of the opacity when the high amount of SiO_2_ was incorporated, it is hard to observe the electrical tree. The electrical tree breakdown time is used to assess the electrical treeing speed of the composites. As shown in Figure 8, the Weibull breakdown time of EP, C10-D20 and C10-D40 are 2.8, 4.1 and 0.8 min. The 40 wt % HSM will increase the treeing speed, which is consistent with the results in Figure 5e. Actually, due to the increasing amount of the SiO_2_ particles, the epoxy:HSM proportions are no longer 8:2 nor 6:4. Then, some adjustments are made by slightly reducing the amount of the SiO_2_ and HSM. In C8-D18 and C6-D38, the epoxy/HSM proportions are still close to 8:2 and 6:4. The Weibull breakdown time of the epoxy/HSM/SiO_2_ composite C8-D18 is 6.6 min, which is 2.4 times that of EP.

### 3.4. Discussion

**Figure 9:**
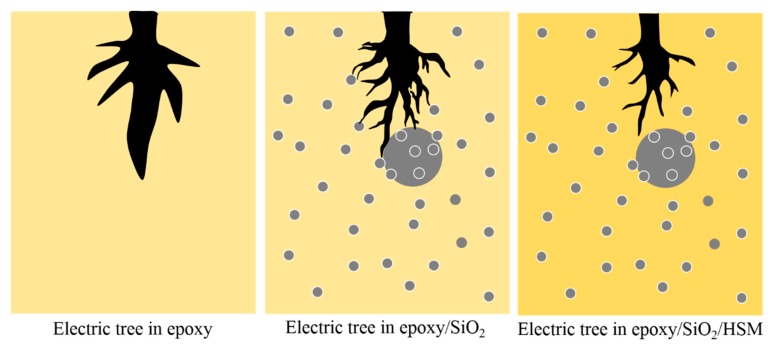
The effects of SiO_2_ and HSM on electrical treeing.

As shown in Figure 9, micro particles have blocking effects on electrical treeing because of the bigger scale than the tree channel. It can be concluded that the inhibiting effects of the nano particles are due to the large number of polymer-particle interfaces which inhibit the charges injection and movement by enhancing the interfacial polarizations and increasing the polymer active energy and the average trap depth. Thus, both incorporation of the micro particles and the nano particles will increase the resistance to the electrical tree. HSM introduces abundant hydrogen bonds into the epoxy matrix, when the local electric field becomes too strong with the development of electric trees, the hydrogen bonds are broken but soon healed, because the increase of local temperate leads to the expansion of the composite and the distance is close enough to form hydrogen bonds again. Thus, the electric trees develop slowly and the treeing channels are thinner.

## 4. Conclusions

Moderate SiO_2_ and HSM may both play a positive role in inhibiting the electrical tree. Besides the self-healing behavior at the macro level, the incorporation of HSM can also make the micro defects such as electrical tree channel self-healable. The self-healing cycles of the epoxy/HSM/SiO_2_ composites increase with the increasing amount of HSM. However, the limited self-healing times of the electrical tree are not caused by the decrease of the electrical property, but the loss of the resistance to the local mechanical stress produced at the tree channel. This research provides a strategy to improve the durability and reliability of polymer dielectrics and extend the lifetime of electrical equipment and electronic devices. The proposed electrical self-healing epoxy composite could be applicable for other types of self-healing materials and polymer dielectrics. Therefore, the electrical self-healing composite will find a wide application in the field of electronic and electrical engineering.

## Supplementary Materials

The following are available online at www.mdpi.com/2073-4360/9/9/431/s1.
