# Supplementary material for: A Self-Healing and Electrical-Tree-Inhibiting Epoxy Composite with Hydrogen-Bonds and SiO2 Particles
Source: Polymers (Basel). 2017 Sep 8;9(9):431. doi: 10.3390/polym9090431 (PMC6418971; doi:10.3390/polym9090431)
Supplement: Supplementary file 1 [file polymers-09-00431-s001.pdf]

A Self-healing and Electrical-tree-inhibiting Epoxy Composite with Hydrogen-bonds and SiO_2 Particles

Supplementary Information

Wancong Bian[†], Wenxuan Wang[†], Ying Yang[*]

[†] These authors contributed equally to this work and should be considered co-first authors.

[*]Correspondence to:

yingyang@tsinghua.edu.cn

Department of Electrical Engineering, Tsinghua University, Beijing 100084, China

1. Dimensions of the samples for electrical treeing experiments.

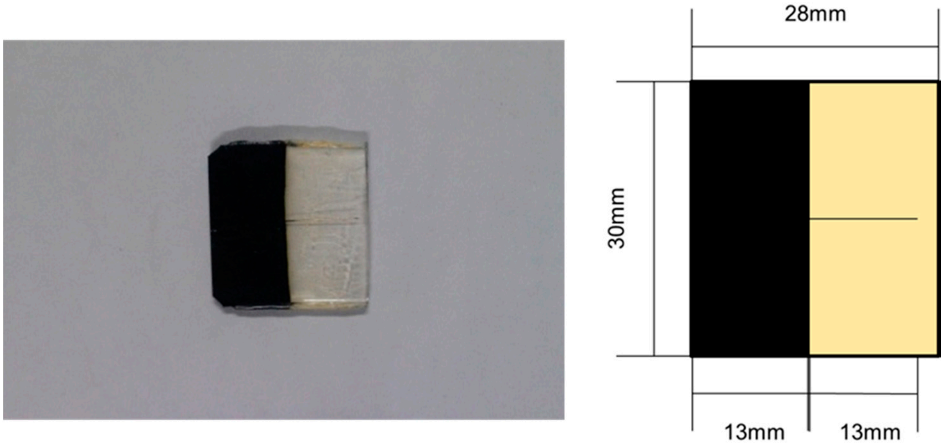

Figure S1. The sample for electrical treeing experiments.

A piece of black conductive rubber was penetrated by a 0.16 mm diameter needle electrode and embedded at one side of the sample to provide a conductive contact with the external electrode. The tip of the needle electrode was located near the other side of the 30×28×3 mm cubic sample. The distance between the tip and the grounding surface is 2 mm (1 mm for electrical tree breakdown testing).

2. Weibull distribution.

In this research, the conventional two-parameter Weibull function was used to analyze the breakdown time of the composites. The cumulative probability P of the electrical failure time is given by:

$$P(t;\alpha,\beta) = 1 - \exp\left\{-\left(\frac{t}{\alpha}\right)^{\beta}\right\} \quad (1)$$

where t is the breakdown time of the sample, β is a shape parameter and α is a scale parameter that indicates the breakdown time at the cumulative probability of 63.2%.

Equation (1) can be also written as:

$$\log(-\ln(1-P)) = \beta\log(t) - \beta\log(\alpha) \quad (2)$$

Thus, the Weibull plot has the relationship shown above between the cumulative probability P and the breakdown time t. Several approximations for cumulative probability P have been proposed, the most popular of which is the Ross function and is given by the following equation:

$$P(i,n) \approx \frac{i-0.44}{n+0.25} \times 100\% \quad (3)$$

where i is the ith and n is the total number of each kind of samples. Using Equations (2) and (3), the values of β and α for different samples can be obtained. It is widely accepted that the α is used to estimate the value of the breakdown time.

3. Chemical structures of the HSM D34 and D45

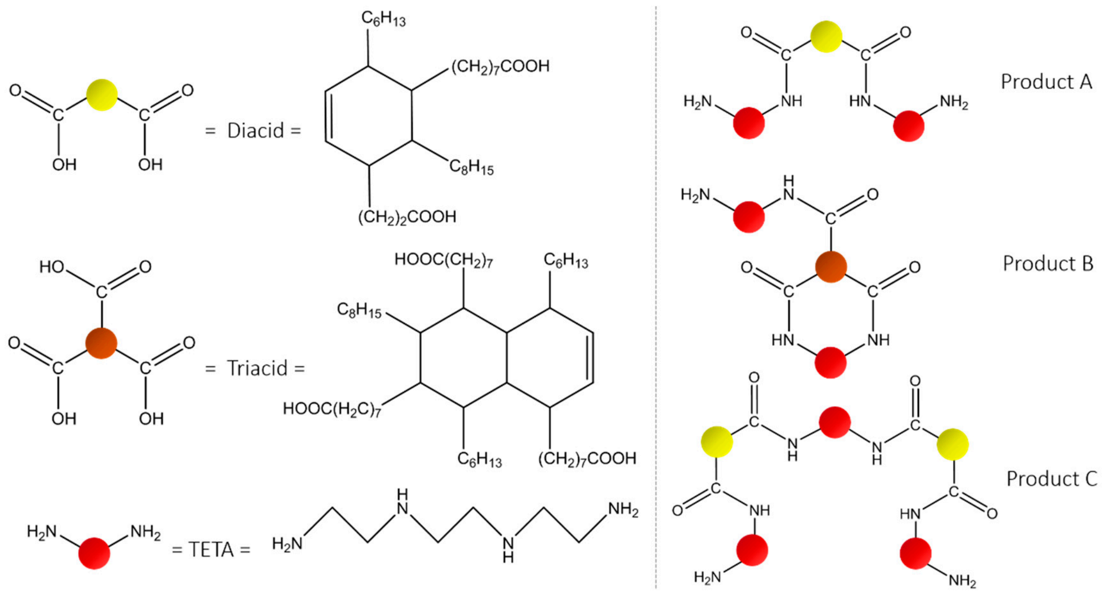

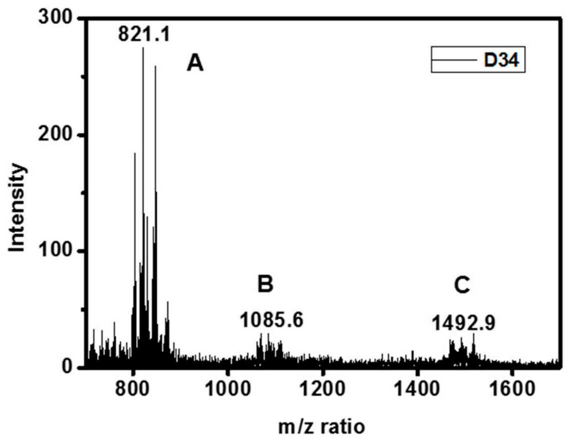

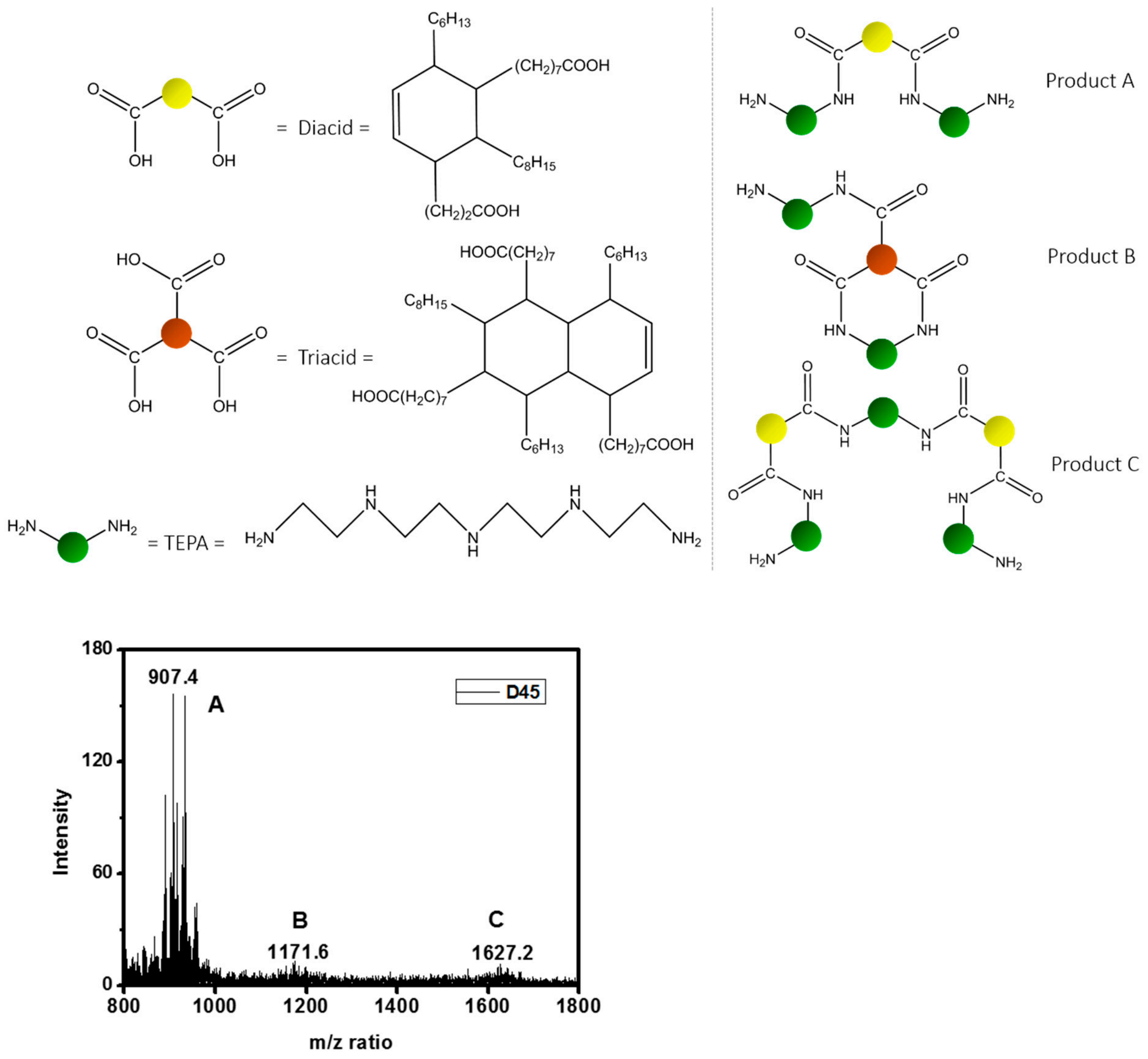

Figure S2. (a) Chemical structures of the three main products A, B and C in the HSM D34; (b) The MS of the products in the D34; (c) Chemical structures of the three main products A, B and C in the HSM D45; (b) The MS of the products in the D45.

3. Dielectric parameters measurement.

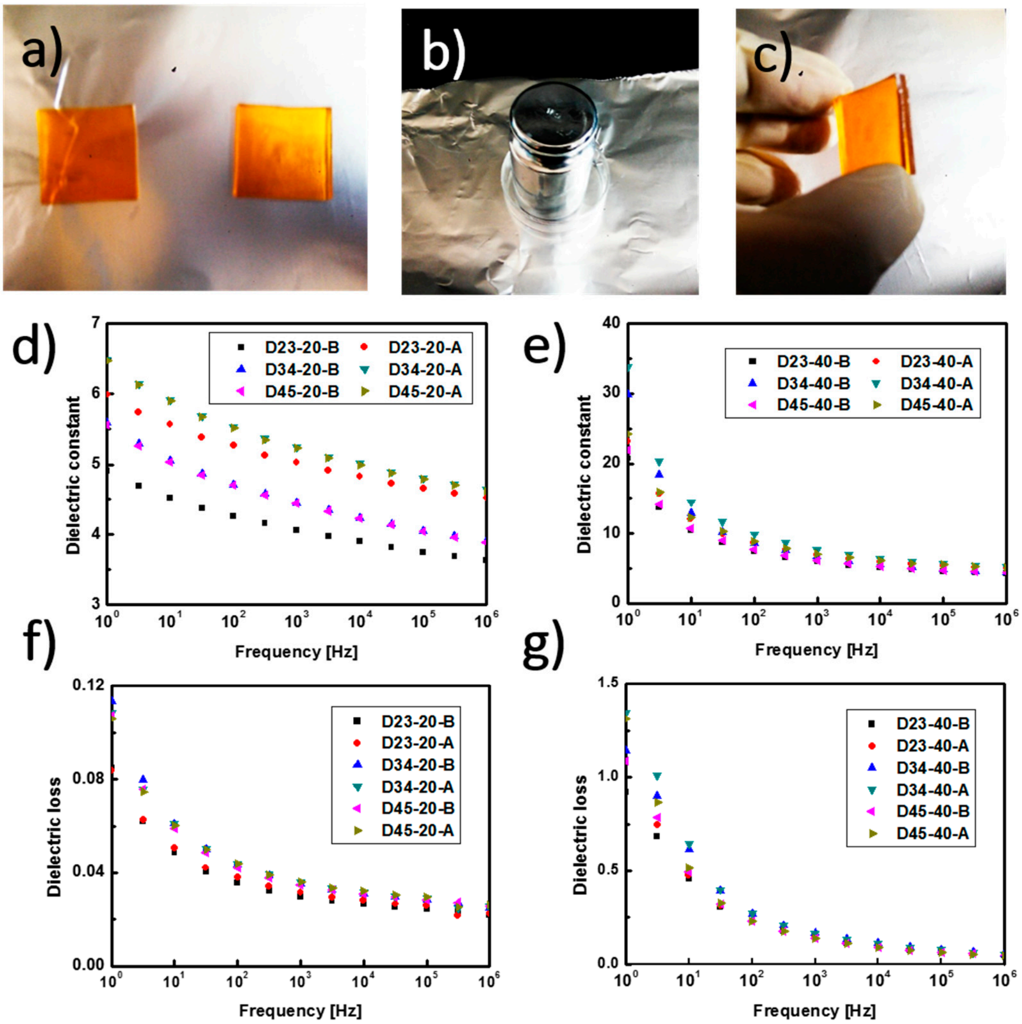

Figure S3. (a) Two separate sheets; (b) Cooling procedure after heating; (c) Two sheets stick to each other after self-healing; The dielectric constants of epoxy/HSM composites with (d) 20 wt.% HSM

and (e) 40 wt.% HSM; The dielectric losses of epoxy/HSM composites with (f) 20 wt.% HSM and (g) 40 wt.% HSM.

4. Differential Scanning Calorimetry Analysis

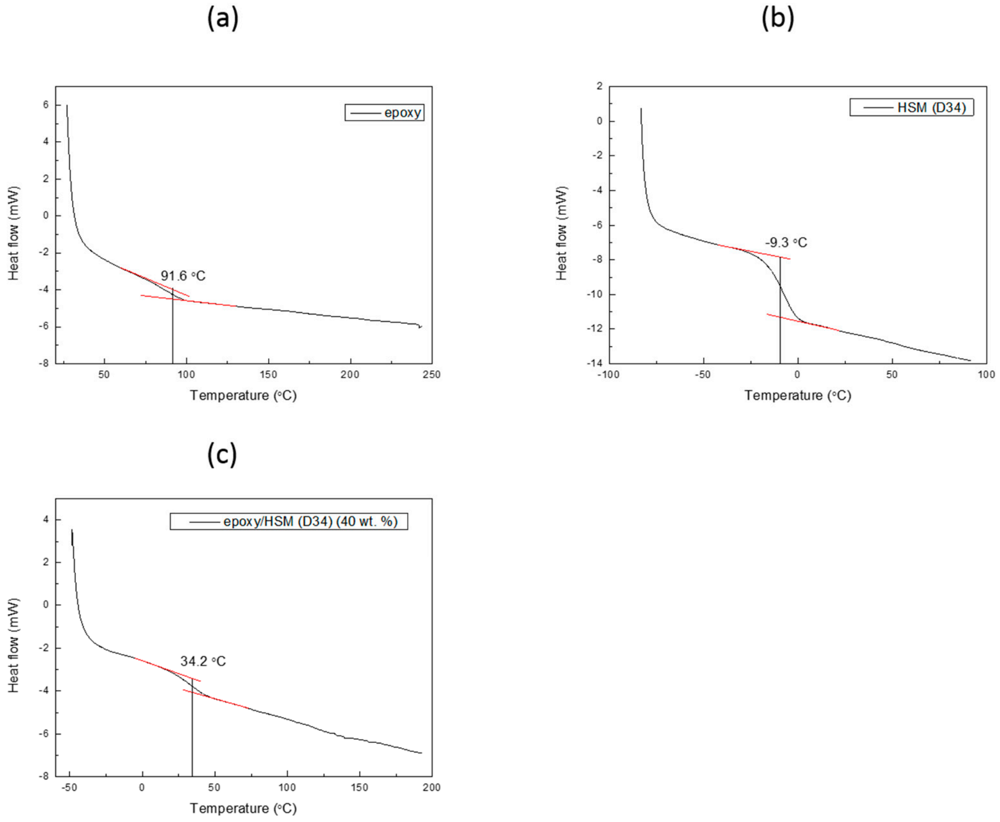

Figure S4. Differential scanning calorimetry (DSC) traces of the (a) epoxy; (b) HSM D34; (c) epoxy/HSM(D34)(40 wt. %).

(a) The glass transition temperature (T_g) of the epoxy is determined to be 91.6 °C.

(b) The T_g of the HSM D34 is is determined to be -9.3 °C.

(c) Only one glass-transition point at 34.2 ℃ was observed in the DSC curve of the epoxy/HSM(D34)(40 wt. %).

All the experiments were conducted at a heating rate of 20 °C/min.

5. An example of the electrical tree path in the epoxy/HSM/SiO2 composite C0.1-D20

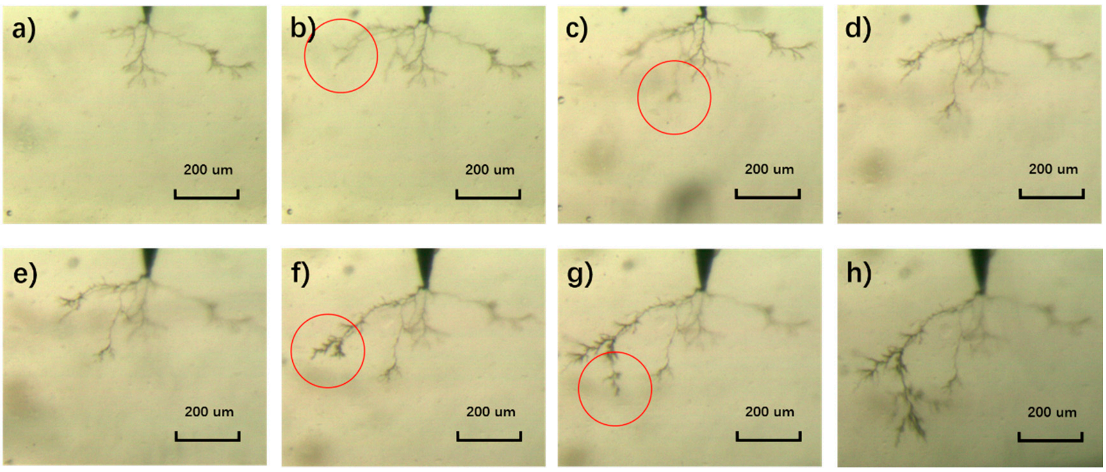

Figure S5. An example of the electrical tree path in the epoxy/HSM/SiO_2 composite: (a) the original electrical tree and (b)~(h) the electrical trees after 1~7 times re-treeing.

Figure S5 gives an example of the electrical tree path in the epoxy/HSM/SiO_2 composite C0.1-D20. The original tree is shown in Figure S5a and the trees after 1~7 times re-treeing are shown in Figure S5b~h. The trees after re-treeing show apparent behavior of growing in a new path rather than extending along the old way as shown in Figure S5b, c, f and g. During the self-healing treatment, the tree channel shrinks as shown in Figure S5g. The hydrogen bonds break, exchange and

recombine, which makes the electrical tree channel to be healed at the micro interfaces. While re-treeing, if the tree tends to grow along the old way, the electrical energy need to be injected to open the healed tree channel once again. In this case, there may not be more advantages for the electrical tree to grow along the original path than find a new path to propagate, which can lead to the time extension for tree reaching another side.

6. Self-healing ability

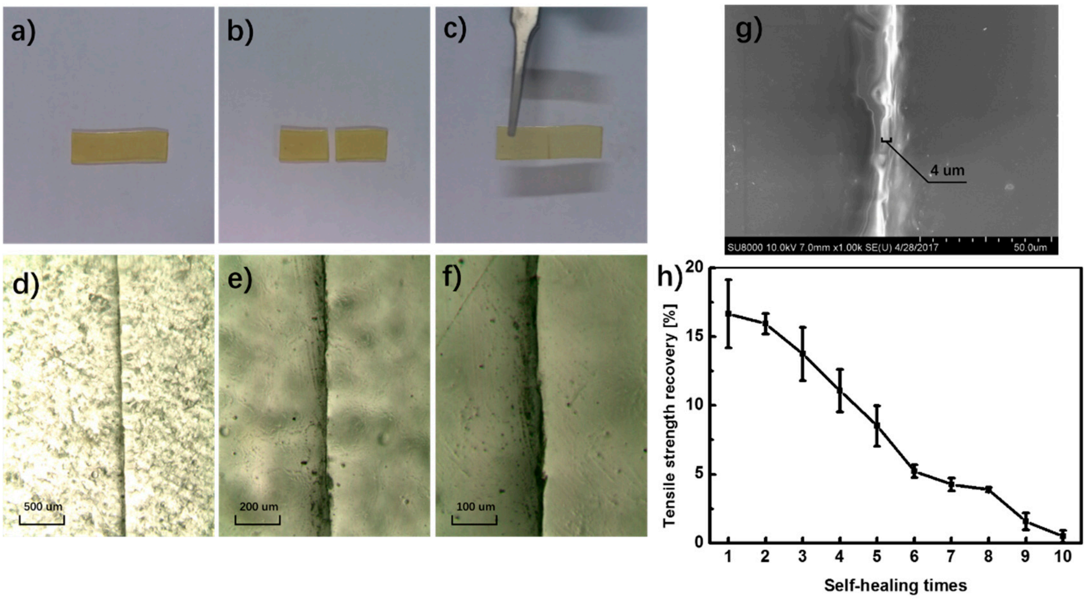

Figure S6. (a) The C0.1-D40 sample prepared as an elongated shape; (b) The sample cut into two separate pieces; (c) Two pieces are sticking together after self-healing at 80 ºC for 2 h; (d)~(g) The microscopic images at the incision; (h) The tensile strength recovery percentage after different self-healing times.

During the degradation of the insulation material while treeing, high-pressure gases are produced [1] and the material is torn apart gradually to form the electrical tree channel. To further simulate

and confirm the reversible self-healing ability of the tree channel interfaces, the tensile strength recovery of the composites after different self-healing times is tested. As shown in Figure S6a~c, the broken sample pieces can reunite as a whole after self-healing. While observing the healed sample under a microscope, no apparent gap is found as shown in Figure S6d~f with different magnifying powers. The SEM image Figure S6g gives the connection width of the transition part between the two pieces as about 4 μm. As shown in Figure S6h, the tensile strength recovery of the epoxy/HSM/SiO_2 at the 1st time is 17% and decreases with the further self-healing cycles. After the 10th self-healing, the recovery percentage is nearly 0%. It indicates that the limited self-healing cycles of the electrical tree are mainly due to the loss of the resistance to the local mechanical stress produced at the tree channel.

7. The hardness of the composites

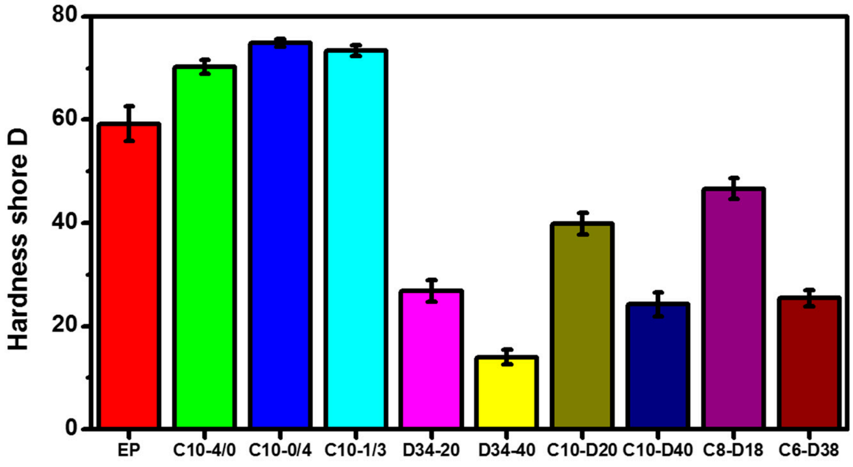

Figure S7. The hardness of the composites.

The breakdown time of the epoxy/HSM/SiO_2 composites are found to connect to the hardness. As shown in Figure S7, the hardness of the C8-D18 is 46.6, which is the highest among the epoxy/HSM/SiO_2 composites. While comparing the hardness of the epoxy/SiO_2 composites and epoxy/HSM composites with the neat epoxy, it can be found that the incorporated HSM decreases the hardness of the composite but the incorporated SiO_2 increases it. It should be noticed that

although the electrical treeing speed is influenced by the mechanical properties, the blocking effect and the inhibition effect of the SiO_2 also play a part in the growing process of the electrical tree.

8. The TGA curves, volume resistivity and trap level density distribution of the composites.

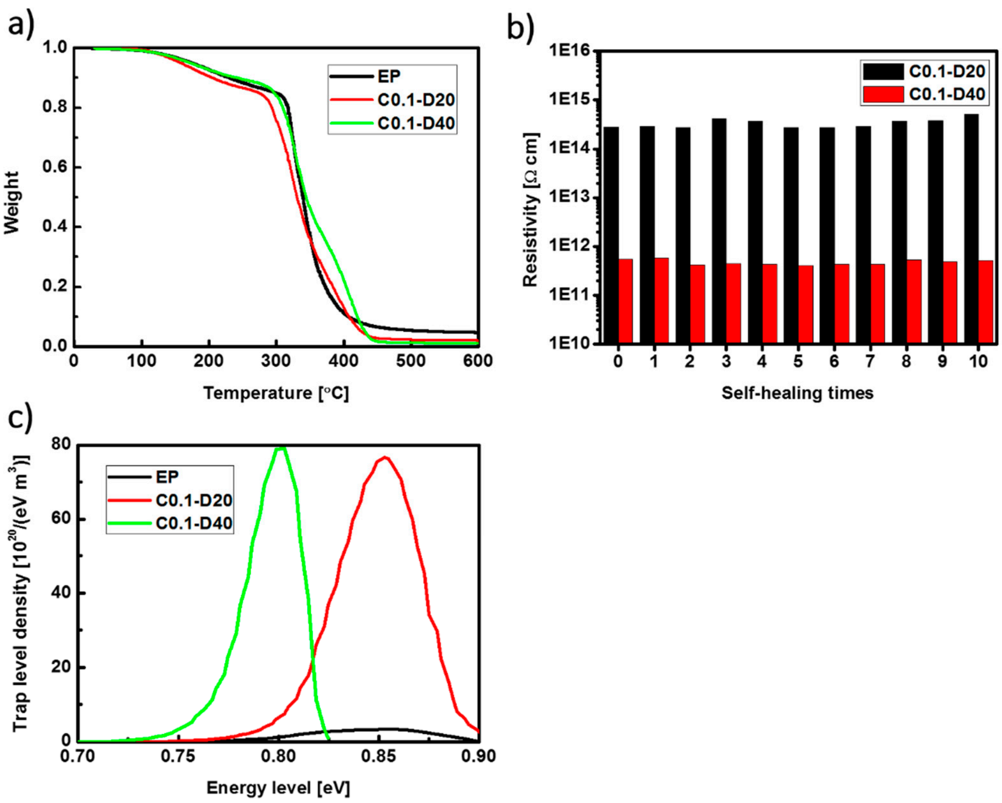

Figure S8. (a) The TGA curves of the composites; (b) The volume resistivity of the composites; (c) The trap level density distribution of the composites.

The formation of the electrical trees accompanies with the degradation of the insulation material.

TGA is used to assess the thermal stability and charring [2] of the composites. As shown in Figure S8a, the degradation temperatures of the EP, C0.1-D20 and C0.1-D40 are 320 °C, 290 °C and 300 °C. The degradation temperatures of the composites are 20~30 °C lower than that of the epoxy resin. Some of the amides in the HSM may react with the epoxy group, forming weaker bonds between the HSM and the epoxy matrix. These bonds rupture first at 290~300 °C, causing the loss of some small molecules formed between the HSM and the epoxy resin. The early decomposition of the small molecules begins at 120 °C in the epoxy resin and the composites. Most insulating materials are used below 100 °C, so the influence of the incorporated HSM/SiO_2 for normal working conditions will be negligible. To confirm the reversible self-healing ability of the tree channel interfaces, the simulated experiments are conducted by measuring the volume resistivity of the composites after several times of self-healing. The neat epoxy shows no self-healing ability and has a resistivity of 10^{14}~10^{15} Ω cm. The original volume resistivities of C0.1-D20 and C0.1-D40 are 10^{14}~10^{15} Ω cm and 10^{11}~10^{12} Ω cm. Figure S8c shows the trap level density distributions of the composites obtained by numerical calculation method [3] according to TSC curves. The trap energy level peaks of the EP, C0.1-D20 and C0.1-D40 are 0.86 eV, 0.85 eV and 0.80 eV. The shallower traps will facilitate the movement and the transport of the charge carriers, which lowers the volume resistivity of C0.1-D40. The volume resistivities of C0.1-D20 and C0.1-D40 are still stable at ~10^{14} Ω cm and ~10^{11} Ω cm after 10 times of self-healing.

References

[1] Yu, S, Ying, L, Linjie, W, Yang, X, Lisheng, Z. Effect of Treeing Test Condition On Voltage Endurance Coefficient for XLPE Cable Insulation. *2015 IEEE 11th International Conference on*

the Properties and Applications of Dielectric Materials: Sydney, Australia. IEEE. (July 2015).

[2] Menard, R, Negrell, C, Ferry, L, Sonnier, R, David, G, Synthesis of Biobased Phosphorus-Containing Flame Retardants for Epoxy Thermosets Comparison of Additive and Reactive Approaches. *Polym. Degrad. Stabil.* **2015**, *120*, 300-312.

[3] Tian, F. *et al.* Theory of modified thermally stimulated current and direct determination of trap level distribution. *J. Electrostat*. **2011**, 69, 7-10.
